# The Twang of the Yankee

**Published:** 1899-07-15

**Authors:** 


### THE TWANG OF THE YANKEE.

A somewhat interesting discussion took place at the meeting of the American Laryngological Association at Chicago in regard to the cause of the "so-called American voice." Many suggestions were made, only to be as quickly controverted, and we fancy it will be found in the end that it is a trick, a habit which having been originally implanted as the result of some accidental circumstance, encouraged maybe by mixture of race and of tongue, has been perpetuated by mere imitation, or perhaps by that curious tendency to survival which seems inherent in tricks, which in certain states of society have some utility. We do not pretend that the American twang is beautiful, but that it has its uses we can hardly deny. It not only is peculiarly fitted for the emphasising of smart sayings, but it is strongly self-assertive, an important virtue at this end of the century. Even amid a crowd of big and burly British voices the keen twang of the Yankee reaches to the end of the room, and from the tendency which is so often shown by those who associate much with Americans, or who make even a short visit to the States to pick it up, and to come back distinctly Americanised in voice, we fancy that in the incisiveness of the twang there is hidden a "fitness" which makes it "survive." One speaker attributed the peculiar voice in question to peculiar abnormalities in the anterior part of the nasal canal, including chronic catarrhal inflammation of the nasal mucosa even without hypertrophy, from all of which causes there resulted anæsthesia and paresis of the soft palate, in consequence of which some tones which should be formed in the mouth were produced in the naso-pharynx at a higher level. But, on considering the ages at which the various causes mentioned were most prevalent, it became clear that the nose alone did not determine the nasal voice, and that in children this was often a mere result of imitation, and was to be cured by placing them among people who speak properly. Another speaker thought the excessive tension of American life was partly the cause of the nasal voice, and that they must learn to cultivate the teachings of the "gospel of relaxation," but he agreed that so far as the anatomical factor was concerned the low-hanging palate during speech was probably the prime element. Again, another speaker attributed to the noise of American cities the high-pitched voice which was necessary to make oneself heard; but, in answer to this, it was pointed out that some of the worst examples are to be found in quiet villages. What is pretty clear, however, is that whether its origin was pathological or racial, and whether or not it possessed certain advantages in a more or less uncouth state of society, its continuance is not to be desired. There are plenty of educated Americans who do not show a trace of the twang, even though they may to the full make use of what we look upon as American expressions and forms of speech, and at the present day we must regard the "so-called American voice" as a mere provincialism, perpetuated by dint of the tendency always so marked among the young not only to imitate but to exaggerate any marked peculiarity among their elders.

